# Exercise training reduces circulating cytokines in male patients with coronary artery disease and type 2 diabetes: A pilot study

**DOI:** 10.14814/phy2.15634

**Published:** 2023-03-11

**Authors:** Léa Garneau, Tasuku Terada, Matheus Mistura, Erin E. Mulvihill, Jennifer L. Reed, Céline Aguer

**Affiliations:** ^1^ Institut du Savoir Montfort – Recherche Ontario Ottawa Canada; ^2^ Department of Biochemistry, Microbiology and Immunology, Faculty of Medicine University of Ottawa Ottawa Ontario Canada; ^3^ Exercise Physiology and Cardiovascular Health Lab University of Ottawa Heart Institute Ottawa Ontario Canada; ^4^ Division of Cardiac Prevention and Rehabilitation University of Ottawa Heart Institute Ottawa Ontario Canada; ^5^ Energy Substrate Metabolism Research Lab University of Ottawa Heart Institute Ottawa Ontario Canada; ^6^ School of Human Kinetics, Faculty of Health Sciences University of Ottawa Ottawa Ontario Canada; ^7^ School of Epidemiology and Public Health, Faculty of Medicine University of Ottawa Ottawa Ontario Canada; ^8^ Department of Physiology, Faculty of Medicine and Health Sciences McGill University Montreal Quebec Canada; ^9^ Interdisciplinary School of Health Sciences, Faculty of Health Sciences University of Ottawa Ottawa Ontario Canada

**Keywords:** coronary artery disease, cytokines, exercise, interleukins, type 2 diabetes

## Abstract

Low‐grade inflammation is central to coronary artery disease (CAD) and type 2 diabetes (T2D) and is reduced by exercise training. The objective of this study was to compare the anti‐inflammatory potential of moderate‐to‐vigorous intensity continuous training (MICT) and high‐intensity interval training (HIIT) in patients with CAD with or without T2D. The design and setting of this study is based on a secondary analysis of registered randomized clinical trial NCT02765568. Male patients with CAD were randomly assigned to either MICT or HIIT, with subgroups divided according to T2D status (non‐T2D‐HIIT *n* = 14 and non‐T2D‐MICT *n* = 13; T2D‐HIIT *n* = 6 and T2D‐MICT *n* = 5). The intervention was a 12‐week cardiovascular rehabilitation program consisting of either MICT or HIIT (twice weekly sessions) and circulating cytokines measured pre‐ and post‐training as inflammatory markers. The co‐occurrence of CAD and T2D was associated with increased plasma IL‐8 (*p* = 0.0331). There was an interaction between T2D and the effect of the training interventions on plasma FGF21 (*p* = 0.0368) and IL‐6 (*p* = 0.0385), which were further reduced in the T2D groups. An interaction between T2D, training modalities, and the effect of time (*p* = 0.0415) was detected for SPARC, with HIIT increasing circulating concentrations in the control group, while lowering them in the T2D group, and the inverse occurring with MICT. The interventions also reduced plasma FGF21 (*p* = 0.0030), IL‐6 (*p* = 0.0101), IL‐8 (*p* = 0.0087), IL‐10 (*p* < 0.0001), and IL‐18 (*p* = 0.0009) irrespective of training modality or T2D status. HIIT and MICT resulted in similar reductions in circulating cytokines known to be increased in the context of low‐grade inflammation in CAD patients, an effect more pronounced in patients with T2D for FGF21 and IL‐6.

## Graphical abstract

We compared the efficacy of high intensity interval training (HIIT) and moderate intensity continuous training (MICT) in improving low‐grade inflammation in patients with coronary artery disease (CAD) with or without type 2 diabetes (T2D). We observe and report that HIIT and MICT were similarly effective in decreasing the levels of the circulating inflammatory markers in both patients with CAD and patients with CAD and T2D. Furthermore, the training interventions showed a greater effect in the reduction of fibroblast‐growth factor 21 (FGF21) and interleukin‐6 (IL‐6) in patients with CAD and T2D than in patients with CAD alone.

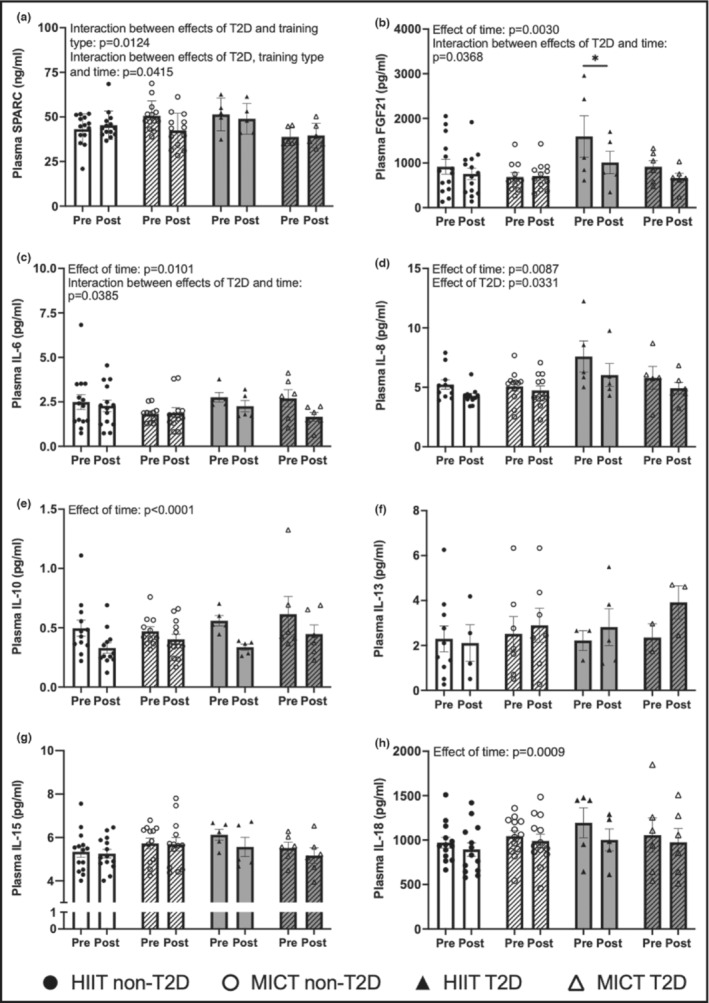

## 1 INTRODUCTION

Coronary artery disease (CAD) is the most common form of heart disease. Following coronary artery revascularization, patients with CAD are referred to cardiovascular rehabilitation programs to improve patients' physical and mental health (Luepker et al., 1996; Mampuya, 2012; Warburton et al., 2007). Conventional cardiovascular rehabilitation programs include moderate‐to‐vigorous intensity continuous training (MICT) to assist patients in attaining weekly exercise recommendations (i.e., 150 min of moderate‐to‐vigorous‐intensity exercise per week in combination with light physical activity; Ross et al., 2020), with at least two sessions per week incorporating exercises to strengthen muscles and bones (resistance training; Tremblay et al., 2011) and improving their cardiovascular health. Type 2 diabetes (T2D) is a non‐communicable chronic disease often associated with a sedentary lifestyle (prolonged sitting time) and/or lack of physical activity, and excess body weight (Booth et al., 2012; Hu et al., 2003; Knowler et al., 2002). T2D is also strongly associated with the development and progression of CAD (Stern, 1995). In a study evaluating the long‐term outcomes of coronary artery bypass graft surgery (CABG) in patients with CAD, the presence of T2D resulted in increased morbidity and mortality over the first 10 years following the surgery, and this increase was exacerbated in patients taking anti‐hyperglycemic medications and insulin (Kogan et al., 2018).

Individuals who suffer from T2D often experience difficulties in engaging in regular exercise due to physical discomforts associated with their disease (e.g., fear of injury, pain related to movement, hypoglycemia, and tiredness), as well as psychological factors affecting their participation in exercise (e.g., depression, shame, lack of motivation, laziness, and fear of the perception of others) (Korkiakangas et al.,  2009). Regardless of existing pathologies, lack of time is frequently cited as a barrier to regular exercise participation and meeting current World Health Organization activity guidelines (i.e., at least 150 min/week of moderate to vigorous aerobic exercise) (Advika et al., 2017). Many researchers have, thus, explored high‐intensity interval training (HIIT) as an alternative to traditional MICT in achieving similar or superior physical and mental health improvements in less time. HIIT has shown promise for the management of T2D as it was shown to induce similar or greater improvements in glucose homeostasis when compared to MICT, even with 45% reduced training volume (Winding et al., 2018).

Atherosclerosis is driven by the accumulation of cholesterol within the arterial wall and chronic non‐resolving inflammation (Libby et al., 2002), which is also an important feature of metabolic syndrome, insulin resistance, and T2D (Hotamisligil, 2006). The state of chronic inflammation induced by imbalances between the regulation of pro‐inflammatory cytokines and chemokines (e.g., interleukin [IL]‐1b, tumor necrosis factor [TNF]‐a, IL‐6 and IL‐8) and anti‐inflammatory cytokines (e.g., IL‐10 and IL‐13) results in impaired glucose and lipid metabolism, tissue dysfunction, and contributes to residual inflammatory risk in cardiovascular death (Hotamisligil, 2017). This underscores the importance of reducing chronic inflammation in patients with CAD with or without T2D. In a recent meta‐analysis examining the effect of exercise interventions (HIIT ~27% of all studies; MICT ~76%; MICT or HIIT in combination with resistance training ~22%) on circulating inflammatory markers in patients with CAD, neither significant reductions in TNF‐a, IL‐6 or IL‐8 concentrations, nor increases in IL‐10 were found; however, a reduction in C‐reactive protein (CRP) suggested diminished acute phase inflammation without changes in chronic inflammatory markers (Thompson et al., 2020). Direct comparisons of the anti‐inflammatory potential of HIIT and MICT are scarce in the literature and both short‐ (2 weeks) (Barry et al., 2018; Robinson et al., 2015) and longer‐term (10–12 weeks) interventions (Bartlett et al., 2017; Mallard et al., 2017) resulted in no changes in circulating inflammatory markers in various populations (healthy individuals, patients with obesity and/or T2D). The exception is one 8‐week protocol of HIIT which led to increased serum CRP and IL‐6, while no significant change was measured with the MICT protocol in the same cohort of overweight or obese participants (Vella et al., 2017). Because most comparisons between exercise modalities are drawn through meta‐analyses with few direct comparisons of HIIT and MICT interventions in clinical populations, the effects of such training on reducing chronic inflammation remain unknown.

The current study assessed the impact of 12 weeks of HIIT‐ or MICT‐based cardiovascular rehabilitation on plasma cytokine concentrations in patients with CAD with or without T2D. We examined 11 target cytokines (IL‐1b, TNF‐a, CRP, secreted protein acidic rich in cysteine [SPARC], fibroblast growth factor 21 [FGF21], IL‐6, IL‐8, IL‐10, IL‐13, IL‐15, and IL‐18) known to be altered in the presence of obesity and/or T2D, and to be regulated by acute and/or chronic exercise (Garneau & Aguer, 2019). It was hypothesized that HIIT and MICT modalities would induce a more significant reduction in circulating cytokines concentrations in the patients with CAD and T2D, as these patients are affected with more pronounced chronic inflammation. As HIIT has been shown to further improve glucose homeostasis than MICT in patients with T2D, we also sought to determine if this training modality would be more effective in reducing inflammatory markers in these patients.

## 2 MATERIALS AND METHODS

### 2.1 Study population

The samples were obtained from the previously published cardiac rehabilitation exercise modalities study (CRX‐Modalities; clinical trial NCT02765568), a randomized clinical trial conducted at the University of Ottawa Heart Institute (UOHI) designed to compare the efficacy of alternative cardiac rehabilitation modalities on short‐ and long‐term physical and mental health outcomes in patients with CAD (Reed et al., 2021). This protocol was approved by the Ottawa Health Sciences Network Research Ethics Board (protocol #: 20160127‐01H). The patients were randomized following the baseline phase in a 1:1:1 ratio between groups in a sex (male vs. female) and age (<60 vs. ≥60 years) stratified manner using a computer‐generated sequence as previously described (Reed et al., 2021). For the current study, only the participants assigned to MICT and HIIT exercise modalities from the original study were included. Participants were assigned to subgroups with or without the comorbidity of T2D (participants without T2D: HIIT‐non‐T2D and MICT‐non‐T2D; patients with T2D: HIIT‐T2D and MICT‐T2D).

The inclusion and exclusion criteria were previously described (Reed et al., 2021), with the exception that only male participants were included in the current study to avoid the confounding effects of unbalanced biological distribution since there were no female participants with T2D. Included participants were patients with CAD aged 40–74 years old who previously underwent a percutaneous coronary intervention or CABG in the previous 4–18 weeks. These patients were also referred to the UOHI cardiovascular rehabilitation program, able to walk autonomously, and willing to attend the on‐site twice weekly cardiovascular rehabilitation program for 12 weeks. Exclusion criteria included: current participation in structured exercise training (>2 days/week), inflammatory disease or active infection, persistent or permanent atrial fibrillation, unstable angina or established diagnosis of chronic obstructive pulmonary disease, severe mitral or aortic stenosis, or hypertrophic obstructive cardiomyopathy, unable to read French or English, or unwilling or unable to return for follow‐up visits at week 12.

### 2.2 Demographic, anthropometric, functional, and metabolic characteristics

Medical information including medications was obtained from clinical databases. At baseline (pre‐) and follow‐up (post‐; within 1 week of completing the 12‐week intervention), the participants' height (baseline measurement only), body mass, waist circumference, body composition (bioelectrical impedance analysis; UM‐041, Tanita, Roxton Industries Inc., Kitchener, ON), resting heart rate (RHR) and blood pressure were measured, and a fasting blood sample was collected to obtain plasma and subsequently measure glucose concentration and glycated hemoglobin levels (HbA1c). A 6‐min walk test (6MWT) was also performed at baseline and follow‐up (Reed et al., 2021). The HIIT participants also underwent a peak graded exercise test on a treadmill to establish peak HR with an electrocardiogram (Way et al., 2020), as is standard practice at the UOHI for higher‐intensity exercise in this patient population.

### 2.3 Exercise interventions

As previously described (Reed et al., 2021), study participants were randomized to MICT or HIIT modalities. Both training protocols were 12 weeks in duration with twice weekly exercise classes performed on‐site at the UOHI. Strength training programs were provided to the participants regardless of group assignment, and they were encouraged to perform one weekly session of strength training exercises on their own (e.g., shoulder press and raise, bent over row, elbow flexion and extension, chest press, squat, lunge, push up, and core exercises). Participants were also instructed to perform 200–400 weekly minutes of moderate‐to‐vigorous aerobic exercise outside their cardiovascular rehabilitation program.

#### 2.3.1 High‐intensity interval training

Classes were 45 min in duration, beginning with 10 min of warm‐up at 60%–70% peak HR, then four training blocks consisting of 4‐min high‐intensity work periods at 85%–95% peak HR interspersed with 3‐min low intensity work periods of at 60%–70% peak HR for a total of 28 min and concluding with 5–10 min of cool‐down at 60%–70% peak HR consisting of strength and stretching exercises. The participants choose to perform the HIIT sessions either on aerobic exercise equipment (treadmill, cycle ergometer, elliptical, etc.) or aerobic dance/movement sequences. HRs were monitored directly on the exercise equipment or with a Polar HR monitor (Polar RS800CX, Polar Electro Oy, Kempele, Finland).

#### 2.3.2 Moderate‐to‐vigorous intensity continuous training

Classes were 1 h in duration, beginning with 10–15 min of walking or low‐intensity use of the exercise equipment as warm‐up, then 10–15 min of continuous aerobic exercise (walking or jogging, cycling, elliptical or rowing) for the first 3 weeks, progressing to 30 min of continuous exercise for the remaining weeks at a HR 20–40 bpm above resting values, and concluding with 15 min of cool‐down consisting of strength and stretching exercises. HRs were monitored using a Polar HR monitor, the participant's own device (e.g., Apple watch) or manual palpation.

### 2.4 Cytokine quantification

The target cytokines were measured in plasma samples collected from the participants at baseline and follow‐up (Week 12) using three single‐plex assays (i.e., CRP U‐plex; SPARC and FGF21 R‐plex) and two multiplex assays (i.e., IL‐1b and TNF‐a U‐plex; IL‐6, IL‐8, IL‐10, IL‐13, IL‐15 and IL‐18 U‐plex) from Meso Scale Discovery (Rockville, MD, USA) following the manufacturer's instructions and as previously published (Garneau et al., 2020). The antibodies in all the assays were validated for target specificity with the exception of SPARC. Information about tested specificity can be found on the datasheet of the U‐plex antibody products (https://www.mesoscale.com/). The intra‐assay coefficient of variation for the standards were: 4.37% for IL‐1b, 4.60% for TNF‐a, 2.28% for CRP, 6.45% for SPARC, 2.85% for FGF21, 4.30% for IL‐6, 3.10% for IL‐8, 4.95% for IL‐10, 6.60% for IL‐13, 5.65% for IL‐15, and 2.95% for IL‐18. The lower and upper limits of detection of each antibody were as follows: 0.227 and 4530 pg/mL for IL‐1b, 0.358 and 2900 pg/mL for TNF‐a, 0.427 and 5780 pg/mL for CRP, 0.443 and 1000 ng/mL for SPARC, 0.58 and 20,000 pg/mL for FGF21, 0.1 and 2060 pg/mL for IL‐6, 0.06 and 2010 for IL‐8, 0.08 and 3610 pg/mL for IL‐10, 2.26 and 2440 for IL‐13, 0.6 and 3000 pg/mL for IL‐15, and 0.28 and 39,100 pg/mL for IL‐18.

### 2.5 Statistical analyses

The age of the participants in the four different groups was compared using two‐way ANOVA, with the factors being type of training and T2D status, and Šidák's multiple comparison was used as a post‐hoc test. Data relating to the characteristics of the participants (BMI, waist circumference, body fat %, systolic and diastolic blood pressure, RHR, fasted blood glucose, glycated hemoglobin (HbA1c) as well as 6MWT results) were first assessed for normality and lognormality in the subgroups using the D'Agostino‐Pearson omnibus K^2^ test. When normality was not achieved, but the data followed a lognormal distribution, they were transformed to their logarithms before further analyses. The data were analyzed by three‐way ANOVA with repeated measures or mixed effects analysis (when time‐points were missing) with the different factors being training type, T2D status and time‐point of intervention (baseline and follow‐up). Šidák's multiple comparisons was used as a post‐hoc test.

Plasma cytokine levels were first analyzed using the ‘identify outliers’ function of Prism 9 with the ROUT method (*Q* = 1%) by subgroup. Any identified outlier was then omitted during subsequent analyses. The remaining data were then assessed for normality and lognormality using the D'Agostino‐Pearson omnibus K^2^ test as previously described, and the necessary transformations were performed. The cytokine concentrations or their logarithm were then analyzed using three‐way ANOVA with repeated measures or mixed effects analysis when data points for pre‐ or post‐training intervention were missing. Šidák's multiple comparisons was used as a post hoc test. When applicable, the transformed variables were used for statistical analyses; however, non‐adjusted values are reported in the results for descriptive purposes. All statistical analyses were performed with the Prism 9 software from GraphPad (San Diego, CA, USA). A *p*‐value <0.05 was considered significant for all tests.

## 3 RESULTS

### 3.1 Impact of the training interventions on participants' characteristics and functional capacity

The number of participants in each group were as follows: *n* = 14 for non‐T2D‐HIIT, *n* = 13 for non‐T2D‐MICT, *n* = 5 for T2D‐HIIT, and *n* = 6 for T2D‐MICT. On average, participants were classified as obese (average BMI: 30.4 kg/m^2^) and were normotensive (average systolic/diastolic blood pressure: 123/79 mmHg) due to medical management. The list of medications taken by the participants is reported in Appendix A.

The anthropometric and metabolic characteristics of the study participants pre‐ and post‐training interventions are presented in Table 1. No significant differences were found in any group at baseline or in response to the training interventions for age, BMI, waist circumference, body fat %, resting systolic or diastolic blood pressure. There was a significant interaction between the effect of time and training modality on changes in systolic blood pressure (*p* = 0.0455), such that values increased in the HIIT groups, but decreased in the MICT groups following the training interventions. We found no effect of time or training modality on fasting blood glucose concentrations, although these were significantly increased in the groups of patients with than without T2D (*p* = 0.0015). As expected, the HbA1c values of both groups with T2D pre‐ and post‐training interventions were higher than those of participants without T2D (*p* < 0.0001), with no significant effect of the training interventions. Following the interventions, regardless of training modality or T2D status, participants' RHR was reduced compared to baseline measurements (*p* = 0.0129) and the distance achieved during the 6MWT increased (*p* < 0.0001).

**TABLE 1:** Characteristics of the study participants pre‐ and post‐training intervention.

	Non‐T2D groups				T2D groups				*p*‐values			*p*‐value interactions			
	HIIT (*n* = 14)		MICT (*n* = 13)		HIIT (*n* = 5)		MICT (*n* = 6)		Effect of time	Effect of training modality	Effect of T2D	Time and training modality	Time and T2D	Training modality and T2D	Time, training modality and T2D
	Pre‐	Post‐	Pre‐	Post‐	Pre‐	Post‐	Pre‐	Post‐							
Age (years)	60.2 ± 7.8		62 ± 7.2		61.8 ± 3.6		56.8 ± 7.5		—	0.4917	0.5410	—	—	0.1987	—
BMI (kg/m^2^)	30.5 ± 5.9	30.2 ± 6.4	29.9 ± 3.4	29.7 ± 3.4	30.9 ± 2.9	31.4 ± 2.7	32.2 ± 10.2	30.6 ± 9.8	0.2400	0.6903	0.6856	0.1113	0.2394	0.8078	0.1903
Waist circumference (cm)	104.1 ± 11.9	104.8 ± 13.7	104.3 ± 8.3	111.1 ± 23.0	107.7 ± 7.6	109.2 ± 7.5	107.9 ± 22.5	105.4 ± 20.9	0.6926	0.7188	0.5141	0.0516	0.7489	0.7136	0.2461
Body fat (%)	28.6 ± 6.7	27.7 ± 5.5	28.4 ± 5.5	28.8 ± 4.7	30.6 ± 4.7	29.8 ± 4.1	28.0 ± 5.9	25.2 ± 4.2	0.1101	0.3776	0.9267	0.6315	0.3106	0.3234	0.3348
Systolic blood pressure (mmHg)	120.3 ± 13.5	123.5 ± 14.6	127.2 ± 14.2	125.1 ± 12.1	116.2 ± 9.8	125.2 ± 20.5	121.5 ± 15.3	115.3 ± 16.3	0.7484	0.9965	0.3800	0.0455	0.7545	0.5190	0.3781
Diastolic blood pressure (mmHg)	79.9 ± 9.0	83.8 ± 10.3	81.3 ± 11.1	81.9 ± 7.5	76.6 ± 6.0	80.2 ± 8.7	76.5 ± 9.4	71.8 ± 10.5	0.6853	0.3847	0.0904	0.1374	0.8070	0.4427	0.6484
Resting heart rate (beats/min)	58.9 ± 8.3	58.1 ± 7.1	62.8 ± 13.6	57.7 ± 10.0	64.6 ± 8.3	58.6 ± 8.6	65.1 ± 10.0	58.5 ± 9.7	0.0129	0.8863	0.5471	0.5946	0.2847	0.7671	0.4291
Fasting blood glucose (mmol/L)	5.23 ± 0.46	5.41 ± 0.51	5.55 ± 0.44	5.20 ± 0.60	5.86 ± 1.39	5.90 ± 1.39	6.37 ± 2.93	7.03 ± 0.47	0.4146	0.1421	0.0015	0.9887	0.2036	0.2055	0.1580
HbA1c (%)	5.75 ± 0.25	5.87 ± 0.31	5.53 ± 0.45	5.72 ± 0.34	6.72 ± 1.54	6.90 ± 1.00	6.85 ± 1.29	6.57 ± 1.09	0.9363	0.6466	<0.0001	0.7247	0.2255	0.6669	0.4785
6‐min walking test (m)	577.8 ± 52.2	620.1 ± 58.4	596.5 ± 64.4	639.1 ± 73.3	533.7 ± 84.3	571.5 ± 73.4	549.4 ± 58.4	617.3 ± 98.4	<0.0001	0.2818	0.0842	0.2334	0.1098	0.1434	0.2246

*Note*: Data are shown as average ± SD. High intensity interval training (HIIT), moderate intensity continuous training (MICT), non‐type 2 diabetes (non‐T2D) groups refers to patients with coronary artery disease (CAD) without T2D and T2D groups refer to patients with CAD and T2D comorbidity, body mass index (BMI), glycated hemoglobin (HbA1c), at baseline (pre‐) and follow‐up (post‐) of a 12‐week training intervention.

### 3.2 Regulation of plasma cytokine levels before and following the training interventions

All cytokines measured were detected in the totality of the samples (HIIT non‐T2D *n* = 14; MICT non‐T2D *n* = 13; HIIT T2D *n* = 5; MICT T2D *n* = 6) except for IL‐13, which was undetected in roughly half of the participants' samples irrespective of group. The process of identification of outliers resulted in the exclusion of certain data points for IL‐1b, TNF‐a, CRP, FGF21, IL‐6, IL‐8, IL‐10, IL‐15, and IL‐18, and all the outliers were found in the non‐T2D groups.

**FIGURE 1:**
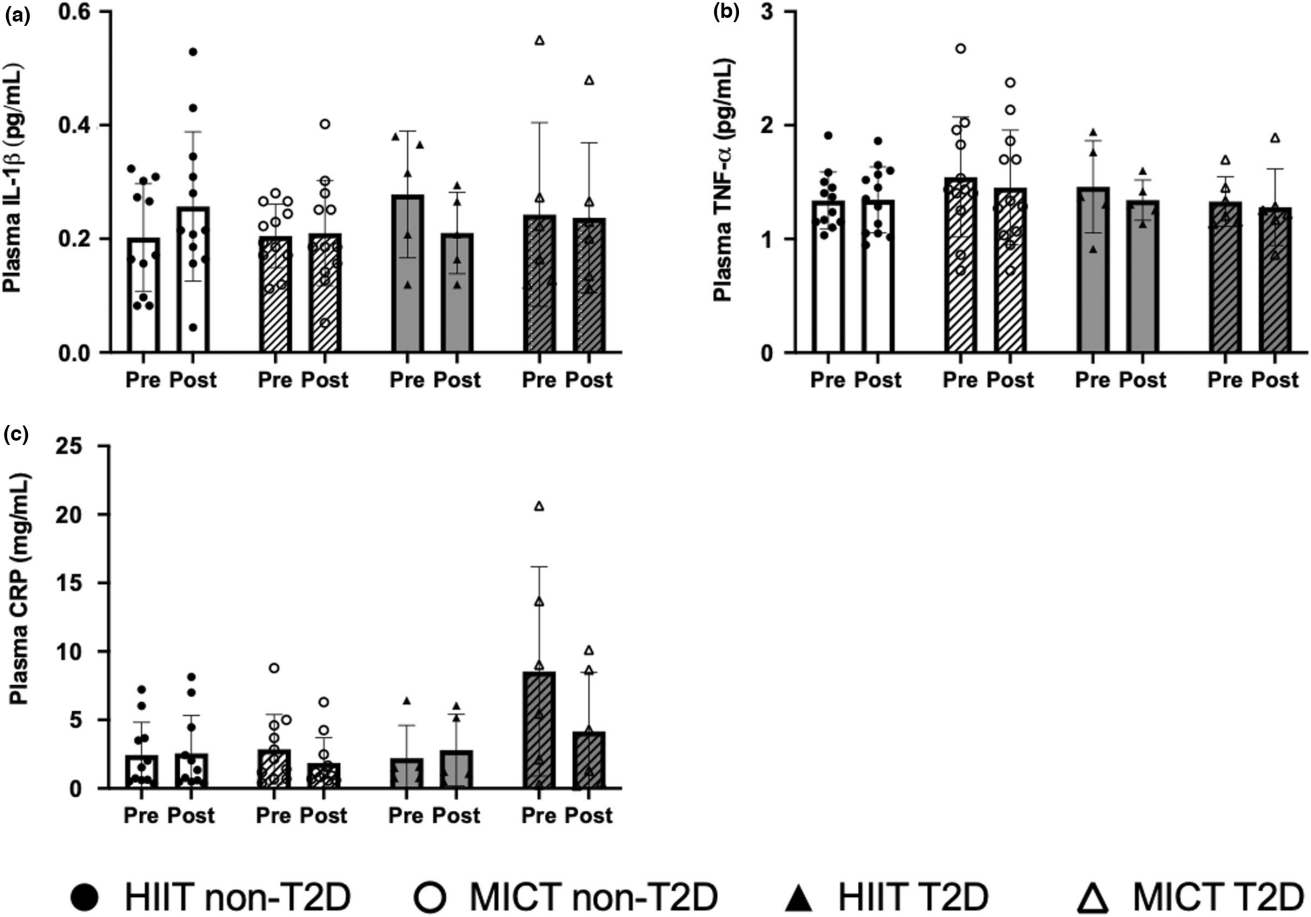
Circulating cytokine concentrations in participants with coronary artery disease (CAD) with or without type 2 diabetes (T2D) in response to a high intensity interval training (HIIT)‐ or moderate intensity continuous training (MICT)‐based rehabilitation program. Plasma cytokine concentrations at rest in men with CAD without (non‐T2D) or with the T2D comorbidity (T2D) before (pre) and after (post) a supervised 12‐week training intervention consisting of either HIIT or MICT. (a) IL‐1β, (b) TNF‐α, and (c) CRP. HIIT non‐T2D group (black circle): *n* = 23 for IL‐1β, *n* = 25 for TNF‐α, *n* = 22 for CRP, MICT non‐T2D group (white circle): *n* = 24 for IL‐1β and TNF‐α, *n* = 22 for CRP, HIIT T2D group (black triangle): *n* = 10 for all, MICT T2D group (white triangle): *n* = 12 for all. Data are shown individually and bars represent the average ± SEM.

**FIGURE 2:**
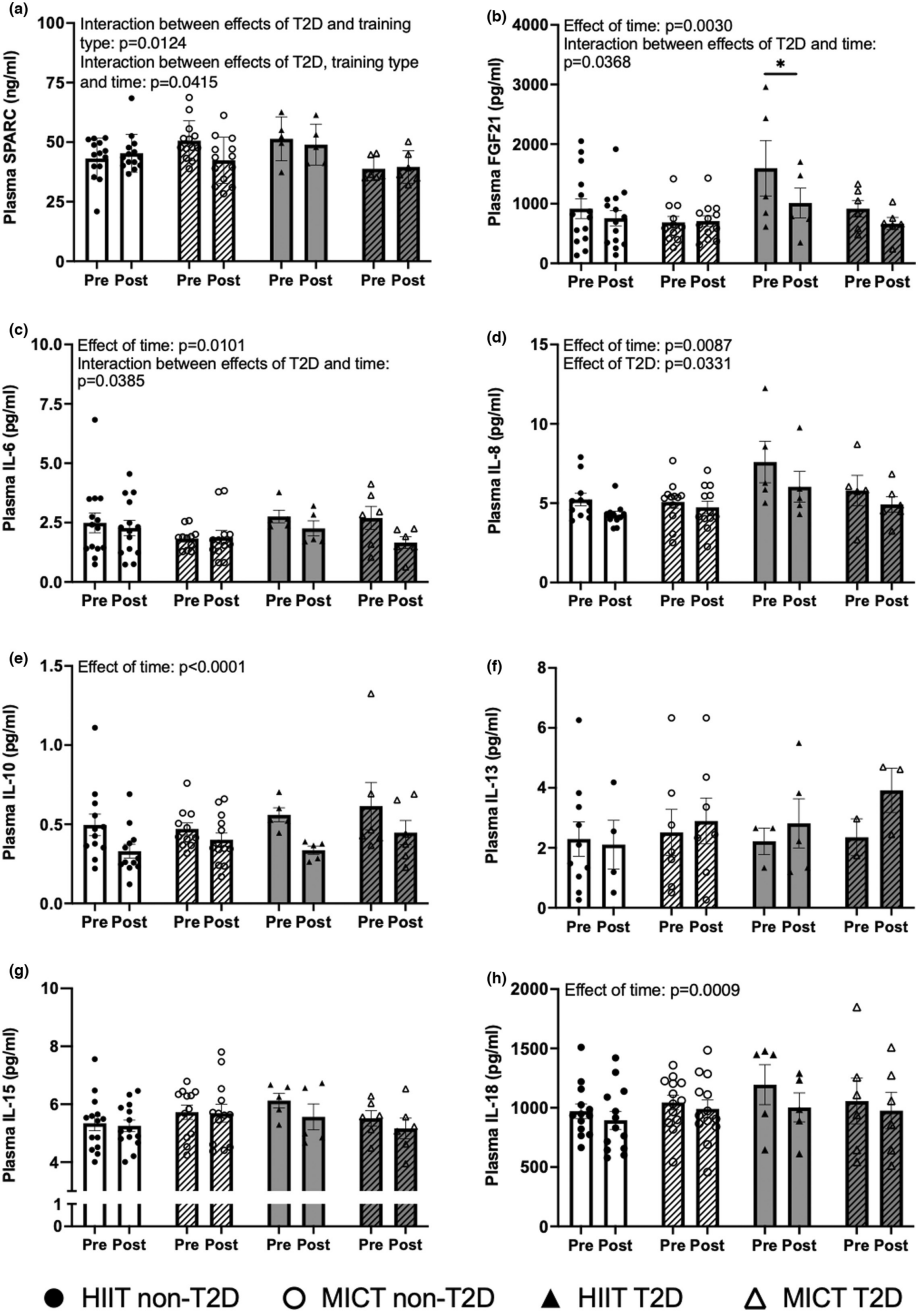
Circulating cytokine concentrations in participants with coronary artery disease (CAD) with or without type 2 diabetes (T2D) in response to a high intensity interval training (HIIT)‐ or moderate intensity continuous training (MICT)‐based rehabilitation program. Plasma cytokine concentrations at rest in men with CAD without (non‐T2D) or with the T2D comorbidity (T2D) before (pre) and after (post) a supervised 12‐week training intervention consisting of either HIIT or MICT. (a) SPARC, (b) FGF21, (c) IL‐6, (d) IL‐8, (e) IL‐10, (f) IL‐13, (g) IL‐15, and (h) IL‐18. HIIT non‐T2D group (black circle): *n* = 28 for SPARC, FGF21, IL‐6 and IL‐15, *n* = 26 for IL‐18, *n* = 24 for IL‐10, *n* = 22 for IL‐8, *n* = 14 for IL‐13. MICT non‐T2D group (white circle): *n* = 26 for SPARC and IL‐18, *n* = 25 for IL‐15, *n* = 24 for IL‐8 and IL‐10, *n* = 23 for FGF21 and IL‐6, *n* = 14 for IL‐13. HIIT T2D group (black triangle): *n* = 10 for SPARC, FGF21, IL‐6, IL‐8, IL‐10, IL‐15 and IL‐18, *n* = 8 for IL‐13. MICT T2D group (white triangle): *n* = 12 for SPARC, FGF21, IL‐6, IL‐10, IL‐15 and IL‐18, *n* = 11 for IL‐8, *n* = 5 for IL‐13. Effect of time refers to differences pre‐ and post‐intervention regardless of training type, type of training effect refers to variations between HIIT and MICT regardless of diabetes status, effect of T2D refers to differences between groups of patients with CAD only or with the comorbidity of T2D. *p < 0.05 post‐ in comparison with pre‐training intervention. Data are shown individually and bars represent the average ± SEM.

We found no effect of time (before and after training interventions consisting of HIIT or MICT) nor of the comorbidity with CAD and T2D on plasma IL‐1b, TNF‐a, and CRP concentrations (Figure 1a–c). Although no effect of time or type of exercise was detected for SPARC, there was a significant interaction between the presence of T2D in the participants and the plasma SPARC levels as a function of training modality (*p* = 0.0124), as well as between T2D, the training modalities and the effect of time (*p* = 0.0415) (Figure 2a). This is portrayed by varying plasma concentrations of SPARC at all time‐points between the groups. The variations in response to training were also different between groups, with an increase in plasma SPARC concentrations in the HIIT‐non‐T2D group and a reduction in the HIIT‐T2D group, whereas the contrary occurred in the two MICT groups. Furthermore, in response to the 12‐week supervised exercise protocols, regardless of the exercise modalities, reductions in resting plasma concentrations of FGF21 (*p* = 0.0030), IL‐6 (*p* = 0.0101), IL‐8 (*p* = 0.0087), IL‐10 (*p* < 0.0001), and IL‐18 (*p* = 0.0009) were observed (Figure 2b,e,h). The reductions in plasma FGF21 in the HIIT‐T2D group (*p* = 0.0499) post‐intervention reached significance in comparison with pre‐intervention concentrations (Figure 2b). An interaction between the co‐occurrence of T2D and CAD in the patients and the changes in plasma concentrations of FGF21 (*p* = 0.0368) and IL‐6 (*p* = 0.0385) in response to training was also detected independently of training modality. The reductions in these cytokines were more pronounced in the groups with T2D. No effects of either HIIT or MICT interventions were detected on resting IL‐13 and IL‐15 plasma concentrations (Figure 2f,g). At all time‐points, plasma IL‐8 concentrations were significantly elevated in the T2D groups in comparison with non‐T2D (*p* = 0.0331) (Figure 2d). No significant effect of T2D was found for any of the other cytokines.

## 4 DISCUSSION

Chronic inflammation is a hallmark of diseases such as CAD and T2D, as elevated concentrations of specific cytokines and other signaling factors are found in the circulation in these patients and often correlate with the severity of their disease (Chen et al., 2007; Trøseid et al., 2009). Regular exercise has conversely been shown to reduce the risk of cardiovascular events through it's influence on improving metabolic parameters (e.g., HbA1c and blood lipid profile) in patients with CAD with or without T2D (Karjalainen et al., 2015). The objective of our study was to compare the anti‐inflammatory potential of traditional (i.e., MICT) and alternative (i.e., HIIT) exercise interventions on the regulation of circulating cytokine concentrations in patients with CAD with or without T2D. We specifically targeted cytokines as markers of chronic inflammation because most of these signaling peptides are increased in the circulation of patients with metabolic syndrome and/or T2D (Hotamisligil, 2006). Consistent with our original hypothesis, the plasma concentrations of certain cytokines (i.e., FGF21 and IL‐6) were further reduced following the training interventions in the groups of patients with T2D. Further, both training modalities yielded significant decreases in plasma cytokine concentrations (FGF21, IL‐6, IL‐8, IL‐10, and IL‐18), suggesting that the 12‐week exercise interventions were sufficient to reduce chronic inflammation in these patients.

### 4.1 The effect of 12 weeks of exercise training on circulating cytokine concentrations

Surprisingly, we found no significant effect of any of the training interventions on plasma concentrations of IL‐1b, TNF‐a and CRP in the patients with CAD with or without T2D. The lack of changes in plasma TNF‐a concentrations following an exercise training intervention in patients with CAD is consistent with the finding of others following a 6‐month HIIT‐based intervention (Munk et al., 2011). However, a 12‐week MICT‐based intervention in CAD patients was found to result in a significant reduction in circulating CRP and IL‐1 concentrations, contrary to what we observed in our patient population (Goldhammer et al., 2005). These differences in findings could be due to several factors, including different assays to measure the cytokines and the presence of angina in their patient population. Regardless, a recent meta‐analysis by Hejazi et al. (2022) showed a consensus in the reductive effect of aerobic exercise training interventions on circulating TNF‐a and CRP concentrations in patients with CAD. Interestingly, their analysis of variations in circulating TNF‐a and CRP concentrations in response to aerobic training interventions showed that patients with a BMI ≥30 kg/m^2^ did not experience significant reductions in either cytokine, while those with BMI 25.0–29.9 kg/m^2^ did not show reduced circulating CRP concentrations following the interventions. On the contrary, a reduction in circulating CRP concentrations was achieved with exercise training in patients with CAD and a BMI <25 kg/m^2^. Consequently, it is possible that the absence of significant changes in circulating levels of these cytokines known to be influenced by exercise levels in patients with CAD could be explained by the demographics of our patient population, since 19 (50%) participants at baseline had a BMI 25.0–29.9 kg/m^2^, 16 (42%) had a BMI ≥30 kg/m^2^, and 3 (8%) had a BMI 20.0–24.9 kg/m^2^. A reduction in circulating FGF21 concentrations (−253.8 pg/mL) was observed across all groups following the 12 weeks of exercise training. Kruse et al. (2017) found that 10 weeks of MICT in patients with T2D did not affect serum concentrations of FGF21. This discrepancy may be due to the medium analyzed (serum versus plasma; as the medium in which cytokines are measured can affect the outcome of interventions on the variation of their circulating concentrations; Lombardi et al., 2017) or the shorter duration of the aerobic exercise bouts in their study (20–35 min). Similarly, across all groups, plasma IL‐6 concentrations (−0.250 pg/mL) were reduced following the exercise intervention. This finding is consistent with those of Sabouri et al. (2021) in a cohort of patients with T2D that includes both sexes following a 12‐week (three sessions per week) HIIT intervention and in patients with CAD following a 12‐week MICT‐based intervention (Goldhammer et al., 2005). A reduction in plasma IL‐8 concentrations was also detected across all of our groups (−0.693 pg/mL). The reductions in both circulating IL‐6 and IL‐8 concentrations are similar to the results of Munk et al. (2011) who measured a reduction in both cytokines in the plasma of patients with CAD following 6 months of HIIT with a similar protocol performed either on a treadmill or cycle ergometer (3 times per week with 4 × 4 min bouts interspersed with 3 min of active rest and some strength and stretching exercises). Plasma IL‐15 concentrations were unchanged following the training interventions (−0.534 pg/mL) irrespective of T2D status (*p* = 0.0562). These findings contrast those of Perez‐Lopez et al. (2018) in healthy and obese individuals, for which habitual physical activity was associated with lower serum concentrations of IL‐15. On the contrary, we expected a reduction in plasma IL‐15 concentrations. It is possible that if our interventions were performed in a larger group of participants, specifically patients with T2D comorbidity, it may have allowed for the detection of significant reductions, as our p‐value was close to significance. Also, we demonstrated that 12 weeks of HIIT or MICT resulted in a reduction in plasma IL‐18 concentrations (−82.19 pg/mL) with no differences between groups regarding T2D status. A previous study revealed no effect of a 12‐month aerobic exercise intervention including HIIT‐based sessions was detected on serum IL‐18 concentrations in a cohort of patients with CAD and T2D composed mostly of men (Zaidi et al., 2019). They also observed no reduction in adipose tissue or leukocyte IL‐18 expression following the training intervention. We did not observe a significant reduction in total body fat percentage following our exercise training interventions. This suggests that the observed reduction in plasma IL‐18 concentrations in response to the 12‐week exercise interventions in the patients with CAD with or without T2D is likely not due to reduced secretion of this cytokine from adipose tissue in accordance with the findings of Zaidi et al. discussed above.

Although we observed a reduction of the anti‐inflammatory cytokine IL‐10 (−0.155 pg/mL) in the plasma of all groups following training, Munk et al. (2011) detected an increase in circulating IL‐10 after 6 months of HIIT and Goldhammer et al. (2005) showed that 12 weeks of MICT in patients with CAD also increased plasma concentrations of this cytokine. Notably, the plasma concentrations measured in these studies were approximately three to fivefold higher than those of our cohort. This could be explained by the duration of the training protocol in the case of the Munk et al. study (i.e., 6 months vs. 12 weeks), the different timing of the blood sample in relation to procedures and intervention, as well as the medical conditions of the patients (i.e., angina vs. CAD without angina), all potentially confounding the comparison between studies. Also, although the kit used to measure IL‐10 by Munk et al. was not described in their methods section, the one used by Goldhammer et al. was a high‐sensitivity quantitative enzyme sandwich immunoassay from a different manufacturer and based on different technology for detection (i.e., colorimetric vs. sulfo‐tag). Nonetheless, it would seem counterintuitive that an anti‐inflammatory cytokine or cytokines that have been shown to have a dual role in inflammatory pathways (i.e., IL‐6 and IL‐18) be diminished in the circulation of patients following a training intervention. An increase in anti‐inflammatory cytokines would be expected. The reduction we observed could be the counterbalance of lower concentrations of pro‐inflammatory cytokines following the training interventions in the patients with CAD.

### 4.2 High‐intensity interval training and moderate‐to‐vigorous intensity continuous training have a similar effect on plasma cytokine concentrations

Few studies have compared the effects of HIIT‐ and MICT‐based exercise interventions on circulating cytokines in clinical populations. In a 12‐month randomized controlled trial of combined aerobic and strength training (three sessions per week) including only patients with T2D, greater reductions in plasma concentrations of IL‐6 were detected following MICT than HIIT in comparison with pre‐intervention values (Magalhaes et al., 2020). The interventions employed by Magalhaes et al. included three sessions of cycling per week matched for energy expenditure between modalities, which gradually increased in intensity up to 40%–60% heart rate reserve (HRR) in the MICT group, and up to 90% HRR for 1‐min bouts interspersed with 1 min of active rest in the HIIT group for a duration matching the prescribed energy expenditure. In the current study, we showed that both training interventions yielded similar reductions in plasma IL‐6 over 12 weeks. The differences between our observations in IL‐6 concentrations in the plasma according to the training modalities could arise from the different types of HIIT protocols employed in both cases, as the intervals in our study were of longer duration and at a lower intensity than those of Magalhaes et al. (2020). Of note, the measured values for circulating IL‐6 in the aforementioned study were approximately 10‐fold higher than ours and most of the data in the literature and their study groups were comprised of men and women in approximately even proportions, while only men were included in our study.

### 4.3 The impact of T2D comorbidity in patients with CAD and their peripheral cytokine levels

We found no differences in plasma SPARC concentrations between patients with or without T2D, while Wang et al. (2015) observed a correlation between serum SPARC concentrations and the homeostatic model assessment of insulin resistance (HOMA‐IR) index in patients with CAD. This discrepancy between the two findings could be due to the medium in which SPARC was measured, as we quantified the cytokine in plasma rather than serum. In patients with CAD, serum FGF21 concentrations are elevated in comparison with healthy subjects and the effect is more pronounced with additional metabolic disorders, such as T2D (Shen et al., 2013). Our findings are not consistent with the literature, as we noted no effect of T2D in increasing plasma FGF21 concentrations compared to patients with CAD alone. On the other hand, there was an additive effect of T2D to the reduction in plasma FGF21 in response to training, which was more pronounced in the patients with T2D. The same interaction between T2D status and the effect of time was found for variations in circulating IL‐6 concentrations. This confirms our research hypothesis regarding these two cytokines as inflammatory markers that can be further reduced by training interventions in patients with both CAD and T2D, rather than CAD alone. In our cohort of patients with CAD, no effect of the T2D comorbidity was detected on plasma IL‐10 and IL‐18 concentrations. In men at risk or diagnosed with CAD, Trøseid et al. (2009) discovered that elevated fasting serum glucose or metabolic syndrome did not affect circulating IL‐10 concentrations. However, they measured higher serum IL‐6 and IL‐18 in participants with poor glucose homeostasis, which positively correlated with their risk of adverse cardiac events. We did not find increased plasma IL‐6 in the groups of patients with T2D, but the reduction in circulating concentrations of this cytokine was greater in patients with T2D in comparison with CAD alone, suggesting an effect of T2D on the response to training. Similarly, Chen et al. (2007) identified a correlation between circulating IL‐18 concentrations and CAD severity. In their study population, the occurrence of T2D positively correlated with elevated IL‐18 concentrations. Contrastingly, Zaidi et al. (2019) revealed no relationship between circulating IL‐18 concentrations and insulin resistance in patients with both CAD and T2D, but a positive correlation between adipose tissue IL‐18 expression and both fasting insulin concentrations and HOMA‐IR values. This finding suggests that elevated IL‐18 concentrations in their patient population might be related to its secretion by adipose tissue. Finally, plasma IL‐8 levels were significantly lower in patients with CAD alone in comparison with patients with T2D (*p* = 0.0331), while Trøseid et al. (2009) demonstrated no effect of metabolic syndrome on IL‐8 circulating concentrations. Of note, their study population consisted of older men (average age: 70 years old) of which approximately one third were current smokers, and cytokines were measured in serum. Any of these factors could explain this contradiction.

### 4.4 Study limitations

Our study design and findings are novel as little information is available in the literature regarding the concentrations of these cytokines in the circulation of patients with CAD following a HIIT or MICT exercise intervention. There are several limitations that warrant mention. First, there is the potential confounding effect of medication between the groups. Indeed, one participant in the MICT non‐T2D group was taking an oral hypo‐glycemic agent at baseline and follow‐up. In healthy individuals, oral hypo‐glycemic agent such as Metformin may influence concentrations of circulating cytokines in a highly variable manner (Ustinova et al.,  2019). One participant in the MICT T2D group also began taking insulin between the baseline and follow‐up visits. The initiation of insulin medication can lead to lipogenic effects in the first 6 months of treatment, leading to macrophage infiltration in sub‐cutaneous adipose tissue and an increase in certain circulating inflammatory factors (e.g., MCP‐1, TNF‐a, and IL‐1b) in patients receptive to these effects of insulin treatment (Jansen et al.,  2013). Second, there were few (*n* = 5 and 6 respectively for HIIT and MICT) participants in the group of patients with the comorbidity of T2D. Because of the smaller sample size across all groups, but specifically for participants with T2D, appropriately powered statistical analyses were not performed. The inconclusive results as pertains to IL‐13 could, therefore, be explained by low statistical power. A more sensitive assay may have yielded more conclusive results for IL‐13 in our patient population. Third, our study did not include any control participants, defined as those not participating in any exercise‐based cardiovascular rehabilitation program. Future investigations should consider the inclusion of (1) patients with CAD yet without T2D and (2) patients with CAD and T2D who did not participate in exercise training interventions to control for the effect of time on potential variations in plasma cytokine concentrations. Without this important control group for reference, there is a possibility that the changes we observed in circulating cytokine concentrations is the result of the natural course of the disease(s). Moreover, our study only included men due to the underrepresentation of women in the subgroups created for this secondary analysis of the original randomized controlled trial (Reed et al., 2021).

Finally, we used plasma as the medium to quantify the cytokines; this component of blood samples prevents the determination of which tissue(s) act(s) as the source of decreased inflammation. Further research into the cytokines measured in this study in response to the two training modalities are warranted to identify their origin (e.g., skeletal muscle, adipose tissue, splanchnic bed, and immune cells) to more thoroughly understand the molecular mechanisms mediating the beneficial metabolic effects of exercise in patients with CAD with or without concurrent T2D.

## 5 CONCLUSIONS

Although our conclusions are limited due to low statistical power in some groups and high inter‐individual variability, these data regarding plasma concentrations of cytokines in patients with CAD and T2D remain of high value for comparison and/or compilation in future studies/meta‐analyses. While we did not find an increased effect of the HIIT‐based exercise intervention program on reducing the cytokines in the circulation of the study participants compared to the MICT‐based intervention, both training types reduced inflammation in these patients. This effect was preserved in patients with both CAD and T2D, and even more pronounced for FGF21 and IL‐6, suggesting that T2D does not interfere with the positive effects of exercise on inflammatory markers. Our findings highlight the potential for both HIIT and MICT to help patients with CAD and co‐morbidities such as T2D to reduce their risk factor of adverse cardiac events by regulating the levels of certain inflammation‐related markers such as the cytokines discussed (i.e., FGF21, IL‐6, IL‐8, IL‐10, and IL‐18).

## AUTHOR CONTRIBUTIONS

Jennifer L. Reed and Céline Aguer conceived and designed research; Jennifer L. Reed and Céline Aguer obtained funding; Léa Garneau and Tasuku Terada performed experiments; Léa Garneau, Tasuku Terada, and Matheus Mistura analyzed data; Léa Garneau, Tasuku Terada, Erin E. Mulvihill, Jennifer L. Reed, and Céline Aguer interpreted results of experiments; Léa Garneau prepared figures; Léa Garneau drafted the manuscript; Léa Garneau, Tasuku Terada, Matheus Mistura, Erin E. Mulvihill, Jennifer L. Reed, and Céline Aguer edited and revised manuscript; Léa Garneau, Tasuku Terada, Matheus Mistura, Erin E. Mulvihill, Jennifer L. Reed, and Céline Aguer approved the final version of the manuscript.

## FUNDING INFORMATION

This clinical trial was funded by the Innovations Fund of the Alternate Funding Plan for the Academic Health Sciences Centres of the Ministry of Ontario (UOH‐16‐003) and the Heart and Stroke Foundation of Canada to J.L.R. Our sub‐study of this trial was supported by the Société Francophone du Diabète to C.A. L.G. was funded by a Ph.D. scholarship from Fonds de Recherche du Québec – Santé.

## CONFLICT OF INTEREST STATEMENT

The authors declare no conflict of interest. The funders had no role in the design of the study; in the collection, analyses, or interpretation of data; in the writing of the manuscript, or in the decision to publish the results.

## ETHICS STATEMENT

The study was conducted in accordance with the Declaration of Helsinki, and approved by the Ottawa Health Science Network Research Ethics Board (protocol #: 20160127‐01H).

## APPENDIX A List of medications taken by the participants at baseline (pre‐) and follow‐up visits (post‐intervention).

### A.1

**Table:**

	Non‐T2D groups *n* (%)				T2D groups *n* (%)			
	HIIT (*n* = 14)		MICT (*n* = 13)		HIIT (*n* = 5)		MICT (*n* = 6)	
	Pre‐	Post‐	Pre‐	Post‐	Pre‐	Post‐	Pre‐	Post‐
ACE inhibitor	7 (50)	7 (50)	5 (38)	5 (38)	2 (40)	1 (20)	4 (67)	4 (67)
Acetylsalicylic acid	13 (93)	13 (93)	10 (77)	10 (77)	3 (60)	3 (60)	5 (83)	5 (83)
Calcium‐antagonist	2 (14)	2 (14)	1 (8)	1 (8)	2 (40)	2 (40)	0 (0)	0 (0)
Statins	12 (86)	12 (86)	11 (85)	11 (85)	5 (100)	5 (100)	6 (100)	6 (100)
Diuretics	1 (7)	1 (7)	1 (8)	1 (8)	0 (0)	1 (20)	0 (0)	0 (0)
Clopidogrel	0 (0)	1 (7)	1 (8)	1 (8)	0 (0)	0 (0)	2 (33)	2 (33)
Nicotine replacement	0 (0)	0 (0)	1 (8)	0 (0)	0 (0)	0 (0)	0 (0)	0 (0)
Anti‐platelet	11 (79)	10 (71)	8 (62)	8 (62)	3 (60)	3 (60)	3 (50)	3 (50)
β‐blocker	12 (86)	11 (79)	8 (62)	9 (69)	5 (100)	5 (100)	5 (83)	5 (83)
Nitrate	6 (43)	6 (43)	8 (62)	8 (62)	2 (40)	2 (40)	3 (50)	3 (50)
Angiotensin receptor blocker	2 (14)	2 (14)	1 (8)	1 (8)	1 (20)	1 (20)	1 (17)	1 (17)
Antidepressant	1 (7)	1 (7)	2 (15)	2 (15)	0 (0)	0 (0)	1 (17)	1 (17)
Insulin	0 (0)	0 (0)	0 (0)	0 (0)	2 (40)	3 (60)	1 (17)	2 (33)
Oral hypoglycemiant	0 (0)	0 (0)	1 (8)	1 (8)	3 (60)	3 (60)	2 (33)	2 (33)
Fibrate	0 (0)	0 (0)	0 (0)	1 (8)	0 (0)	0 (0)	0 (0)	0 (0)
Coumadin	0 (0)	0 (0)	0 (0)	0 (0)	0 (0)	1 (20)	0 (0)	0 (0)

*Note*: High intensity interval training (HIIT), moderate intensity continuous training (MICT), non‐type 2 diabetes (T2D) groups refers to patients with CAD without T2D and T2D groups refer to patients with CAD and this comorbidity, at baseline (pre‐) and follow‐up (post‐) of a 12‐week training intervention.
